# “I’ve discovered the COVID-19 vaccine”. Approach of a bipolar disorder clinical case in the Mental Health Day Hospital of Salamanca during the pandemic

**DOI:** 10.1192/j.eurpsy.2022.1046

**Published:** 2022-09-01

**Authors:** A. Gonzalez-Mota, C. Fombellida Velasco, A. Gonzalez Gil, P. Gómez Hernández, I. Vicente Torres, M. Covacho Gonzalez, C. Payo-Rodriguez, E. Beltran-Mercado, C. Roncero

**Affiliations:** 1 Institute of Biomedicine of Salamanca (IBSAL), Psychiatry, Salamanca, Spain; 2 University of Salamanca Healthcare Complex, Psychiatry, Salamanca, Spain; 3 University of Salamanca, Psychiatry, SALAMANCA, Spain

**Keywords:** bipolar disorder, Psychotherapy

## Abstract

**Introduction:**

A 21-year-old woman diagnosed with bipolar disorder was hospitalized in the Mental Health Day Hospital of Salamanca during the Covid pandemic. The patient engaged with 4 different jobs and a master’s degree, beginning with verbose speech, dysphoria, global insomnia, grandiose delusions, extremely high energy and thinking she has the vaccine. She works the following objectives:illness insight, risk factors, psychopathological stabilization, social skills, slowing down of activities and taking responsibilities.

**Objectives:**

The objective is do a follow-up of the patient during her hospitalization in the Mental Health Day Hospital and to carry out a structured search in PubMed and Up-to-Date about psychotherapy and bipolar disorder.

**Methods:**

3-month follow-up of a 21-year-old woman diagnosed with bipolar disorder during her hospitalization in the Mental Health Day Hospital in Salamanca and a structured search in PubMed and Up-to-Date in April 2021 in English, French and Spanish, including the last 10 years with the keywords “psychotherapy”, “psychotherapies” and “bipolar disorder “.77 studies were analyzed: 12 included, 65 excluded.

**Results:**

Several randomized trials highlight the efficacy of group psychoeducation and cognitive-behavioural therapy in relapse prevention, improving illness insight, medical adherence and less hospitalizations. Therapeutic alliance plays a significant role in the process. Our patient improved her knowledge of her illness and treatment, her social skills and reconnected with her relatives and slowed down her activity. She then was referred to her community mental heath center psychiatrist.

**Conclusions:**

The insight in bipolar disorder plays an important role in medical adherence and prevention of relapses. Therapeutic alliance improves their insight, their functionality in their daily life and enables close monitoring. Medical treatment should be accompanied by psychotherapy for a complete approach of the treatment.

**Disclosure:**

No significant relationships.

